# Cerebrospinal Fluid Flow and Vorticity in Hydrocephalus on 4-Dimensional Flow MRI

**DOI:** 10.1227/neuprac.0000000000000166

**Published:** 2025-10-02

**Authors:** Shigeki Yamada, Ko Okada, Hirotaka Ito, Ryota Fujinami, Tomoyasu Yamanaka, Hiroshi Yamada, Motoki Tanikawa, Chifumi Iseki, Yoshiyuki Watanabe, Satoshi Ii, Tomohiro Otani, Shigeo Wada, Marie Oshima, Mitsuhito Mase

**Affiliations:** * Department of Neurosurgery, Nagoya City University Graduate School of Medical Science, Aichi, Japan;; ‡ Interfaculty Initiative in Information Studies/Institute of Industrial Science, The University of Tokyo, Tokyo, Japan;; § Medical System Research & Development Center, FUJIFILM Corporation, Tokyo, Japan;; ‖ Department of Behavioral Neurology and Cognitive Neuroscience, Tohoku University Graduate School of Medicine, Sendai, Japan;; ¶ Division of Neurology and Clinical Neuroscience, Department of Internal Medicine III, Yamagata University School of Medicine, Yamagata, Japan;; # Department of Radiology, Shiga University of Medical Science, Shiga, Japan;; ** Department of Mechanical Engineering, School of Engineering, Institute of Science Tokyo, Tokyo, Japan;; †† Department of Mechanical Science and Bioengineering, Graduate School of Engineering Science, The University of Osaka, Osaka, Japan

**Keywords:** Flow dynamics, Vorticity, Cerebrospinal fluid, Hydrocephalus, 4D flow MRI, Ventriculoperitoneal shunting, Endoscopic third ventriculostomy

## Abstract

**BACKGROUND AND OBJECTIVES:**

The pathophysiology of obstructive hydrocephalus caused by cerebral aqueduct obstruction or stenosis, as well as communicating hydrocephalus with oscillating cerebrospinal fluid (CSF) flow and vortices in the third ventricle (3V), remains unclear. In addition, clear criteria for selecting between ventriculoperitoneal shunting (VPS) and endoscopic third ventriculostomy (ETV) have yet to be established. This study aimed to investigate the utility of 4-dimensional flow MRI for assessing CSF dynamics to enhance our understanding of hydrocephalus pathophysiology and guide treatment selection.

**METHODS:**

Using 4-dimensional flow MRI (velocity encoding = 5 cm/s), we evaluated CSF dynamics before and after VPS and ETV in patients with obstructive and communicating hydrocephalus, focusing on velocity vectors, streamlines, and vorticity.

**RESULTS:**

In obstructive hydrocephalus due to cerebral aqueduct obstruction, we initially expected a complete absence of CSF circulation in the 3V and cerebral aqueduct before ETV. However, slow flow was observed around the foramen of Monro and even within the obstructed aqueduct. After ETV, rapid CSF flow with vortex formation was observed in the 3V. In obstructive hydrocephalus, while VPS reduced ventricular size, it did not enhance CSF movement in the 3V. In communicating hydrocephalus with third ventricular floor ballooning, rapid CSF flow and abnormal vortices in the 3V persisted after ETV; however, these abnormalities were mitigated by VPS.

**CONCLUSION:**

These findings suggest that both CSF stagnation and abnormal vortices in the 3V contribute to the pathophysiology of obstructive and communicating hydrocephalus. Therefore, treatment strategies should prioritize interventions that optimize CSF flow dynamics.

**ABBREVIATIONS:**
3V: third ventricle
4D: 4-dimensional
4V: fourth ventricle
ETV: endoscopic third ventriculostomy
FOV: field of view
TE: time echo
TR: time repetition
VENC: velocity encoding
VPS: ventriculoperitoneal shunting.

The ventricular system includes the lateral, third, and fourth ventricles (4Vs), with the latter connecting to the subarachnoid space through the tapered structure of the foramina of Magendie and Luschka. In healthy individuals, cerebrospinal fluid (CSF) flows preferentially from the 4V into the posterior subarachnoid space, with the strongest pulsations observed in the cerebral aqueduct, which helps buffer pressure differences between the supratentorial and infratentorial compartments. Under healthy conditions, our previous computational fluid dynamics study,^1,2^ and research using zebrafish models,^3^ have demonstrated that CSF exchange between the third and 4Vs is minimal.

Dynamic imaging techniques such as ultrasound are widely used in clinical practice, particularly in echocardiography, where complex blood flow dynamics, including vorticity and energy loss, are analyzed to assess heart failure severity.^4-6^ However, ultrasound echo is unable to visualize CSF movement within the thick, rigid skull. Recently, 4-dimensional (4D) flow MRI, originally developed for imaging blood flow in the heart and aorta,^7-9^ has gained interest for capturing CSF flow patterns.^10-13^

Hydrocephalus is broadly classified into obstructive hydrocephalus, caused by a blockage of flow in the cerebral aqueduct, and communicating hydrocephalus, in which no blockage is present. Endoscopic third ventriculostomy (ETV) has been reported to be more effective than ventriculoperitoneal shunting (VPS) for obstructive hydrocephalus.^14,15^ For communicating hydrocephalus, some reports suggest that ETV is effective when ballooning of the third ventricular floor is observed^16,17^; however, its effectiveness remains controversial due to unclear underlying mechanisms.^18^ Rather than attributing hydrocephalus solely to obstruction or narrowing of the cerebral aqueduct, we aimed to gain a deeper understanding of CSF dynamics within the ventricular system using 4D flow MRI. This approach may help refine surgical decision-making, guide the selection between ETV and VPS, and predict postoperative outcomes. Inspired by the importance of vortex dynamics in intracardiac blood flow studies,^7^ we aimed to apply quantitative vortex analysis using 4D flow MRI to obstructive and communicating hydrocephalus.

## METHODS

### Ethical Approvals

The study design and protocol of this prospective observational study were approved by the Ethics Committees for Human Research of our institutions (Institutional Review Board number: 60-22-0083, 2022-73, R2019-227). MRI data from patients were obtained using an opt-out method after their personal information had been anonymized in a linkable manner. Written parental consent was obtained for all patients younger than 18 years. Healthy volunteers provided written informed consent after receiving an explanation of the study's purpose and the potential for detecting brain diseases, and subsequently underwent MRI examinations. The study was conducted according to the approved guidelines of the Declaration of Helsinki.

### Study Population

Details of data collection, anonymization, image acquisition, and data processing methods for 4D flow MRI have been described in our previous publications.^11,12,19^ In brief, 226 adult patients diagnosed with chronic hydrocephalus underwent 4D flow MRI between 2017 and 2025 at 3 collaborating hospitals. The present study specifically included patients classified as having midlife hydrocephalus,^20^ who had been excluded from our earlier studies. Patients with other categories of adult chronic hydrocephalus—such as idiopathic normal pressure hydrocephalus, recently renamed Hakim's disease under the new classification,^20^ or secondary normal pressure hydrocephalus—were excluded from the analysis. Midlife hydrocephalus is considerably less common than Hakim's disease in the elderly, making it challenging to collect a large number of comparable cases.

### Image Acquisitions

All MRI examinations were performed with 64-channel 3-T MRI systems made by GE Healthcare, Siemens AG, and Philips at the collaborating hospitals. The 4D flow MRI parameters were as follows: GE: time repetition (TR), variable (10-20 ms); time echo (TE), variable (3-7 ms); flip angle, 8°; field of view (FOV), 200 mm; matrix, 256 × 256; voxel size, 0.781 × 0.781 × 1.0 mm; cardiac phases, 12; velocity encoding (VENC), 5 cm/s. Philips: TR, variable (10-20 ms); TE, variable (3-7 ms); flip angle, 8°; FOV, 200 mm; matrix, 208 × 198; voxel size, 0.89 × 0.89 × 1.0 mm; cardiac phases, 8; VENC, 5 cm/s. Siemens: TR, 100 ms; TE, 9 ms; flip angle, 8°; FOV, 200 mm; matrix, 192 × 154; voxel size, 1.0 × 1.0 × 1.3 mm; cardiac phases, 12; and VENC, 5 cm/s.

Images were acquired in the midsagittal plane over a 30-mm slab (1.0 mm × 30 slices), covering from the bilateral foramina of Monro to the upper cervical subarachnoid space. Imaging time was approximately 10 minutes, depending on heart rate, which was synchronized with peripheral pulse measurements from the finger. Because the 4D flow sequence lacked sufficient resolution in the T1-weighted magnitude images, a 3-dimensional (3D) T2-weighted fast spin-echo sequence was additionally performed to obtain anatomic details. Parameters were: TR, 2000 ms; TE, 85.3 ms; matrix, 288 × 288; voxel size, 0.8 × 0.8 × 0.8 mm; acquisition time, ∼4 minutes.

### Data Processing

The 4D flow application on an independent 3D volume analyzer workstation (SYNAPSE 3D; FUJIFILM Corporation) was approved as a medical device by the Pharmaceuticals and Medical Devices Agency of Japan in 2020. The details of the 4D flow MRI acquisition method have been described in our previousarticles.^11,19,21^ In brief, the first step of 4D flow MRI involves combining 3D velocity-encoding data obtained from triaxial phase-contrast images with morphological data of the intracranial CSF space acquired from 3D T2-weighted MRI using the 4D flow application.

In the second step, the flow velocity measurement region of 4D flow MRI is extracted.

In the third step, automated polynomial fitting is applied to correct for phase offsets, eddy currents, and background noise, ensuring accurate measurement of slow CSF flow velocities.^22^ Finally, 2D and 3D flow velocity vectors, streamlines, wall shear stress, and vorticity can be visualized, as described below. The vorticity measurement technology is available only in the development version of the 4D flow application and has not been approved as a medical device.

### Vorticity Assessment Parameters

To quantify the strength of vortex flow, the vorticity $\vec{\omega }$, which represents rotational flow, is defined as follows:

$$\vec{\omega }=\text{rot}\left(\vec{u}\right)=\nabla \times \vec{u}$$

where $\vec{u}$ (*u*_*x*_, *u*_*y*_, *u*_*z*_) represents the velocity vector at a given time and position.

The Nabla operator, ∇, is expressed as (∂/∂*x*, ∂/∂*y*, ∂/∂*z*).

In a 3D Euclidean coordinate system, the components of the vorticity vector $\vec{\omega }$ are given by:

$$\vec{\omega }=\left(\frac{\partial u_{z}}{\partial y}-\frac{\partial u_{y}}{\partial z},\frac{\partial u_{x}}{\partial z}-\frac{\partial u_{z}}{\partial x},\frac{\partial u_{y}}{\partial x}-\frac{\partial u_{x}}{\partial y}\right)$$

When analyzing a 3D vortex by projecting the velocity vector onto an observed cross-section, the normal component of the vorticity vector was calculated as the rotational speed:

$$\vec{\omega }\cdot \vec{n}=n_{x}\left(\frac{\partial u_{z}}{\partial y}-\frac{\partial u_{y}}{\partial z}\right)+n_{y}\left(\frac{\partial u_{x}}{\partial z}-\frac{\partial u_{z}}{\partial x}\right)+n_{z}\left(\frac{\partial u_{y}}{\partial x}-\frac{\partial u_{x}}{\partial y}\right)$$

where $\vec{n}$ (*n*_*x*_, *n*_*y*_, *n*_*z*_) represents the normal vector of the observed cross-section.

The magnitude of this normal component was maximized when the cross-section was perpendicular to the vorticity vector. The intensity of the displayed color increased accordingly, with clockwise rotation (negative vorticity) shown in blue and counterclockwise rotation (positive vorticity) in red. Vorticity serves as a parameter for evaluating the intensity of local vortex flow; however, it does not directly provide information about the overall macroscopic vortex structure.

## RESULTS

Among 226 adult patients with chronic hydrocephalus who underwent 4D flow MRI, 11 (4.9%) were diagnosed with midlife hydrocephalus. Of these, 6 patients (3 women and 3 men; age range: 23-75 years) were diagnosed with obstructive hydrocephalus, and the remaining 7 patients (6 women and 1 man; age range: 19-71 years) were diagnosed with communicating hydrocephalus. Of the 6 patients with obstructive hydrocephalus, 4 underwent VPS. Among them, 2 patients (including case 1) experienced a rapid worsening of symptoms within a few months postoperatively and required additional ETV, resulting in a challenging clinical course. Of the remaining 2 patients, 1 (case 2) initially underwent ETV and achieved a favorable outcome without the need for further intervention, whereas the other, who presented only with headache symptoms, has been managed conservatively without surgery. Among the 7 patients with communicating hydrocephalus, 1 young patient (case 3) initially underwent ETV and demonstrated temporary improvement; however, symptoms rapidly worsened within a few months, necessitating additional VPS treatment. The other 6 patients (including case 4) initially underwent VPS, and all showed sustained postoperative improvement.

Before presenting the CSF dynamics in 4 instructive cases of midlife hydrocephalus assessed using 4D flow MRI, we first provide an overview of the normal CSF flow pattern in the healthy brain.

### Normal CSF Flow Pattern

**FIGURE 1.**
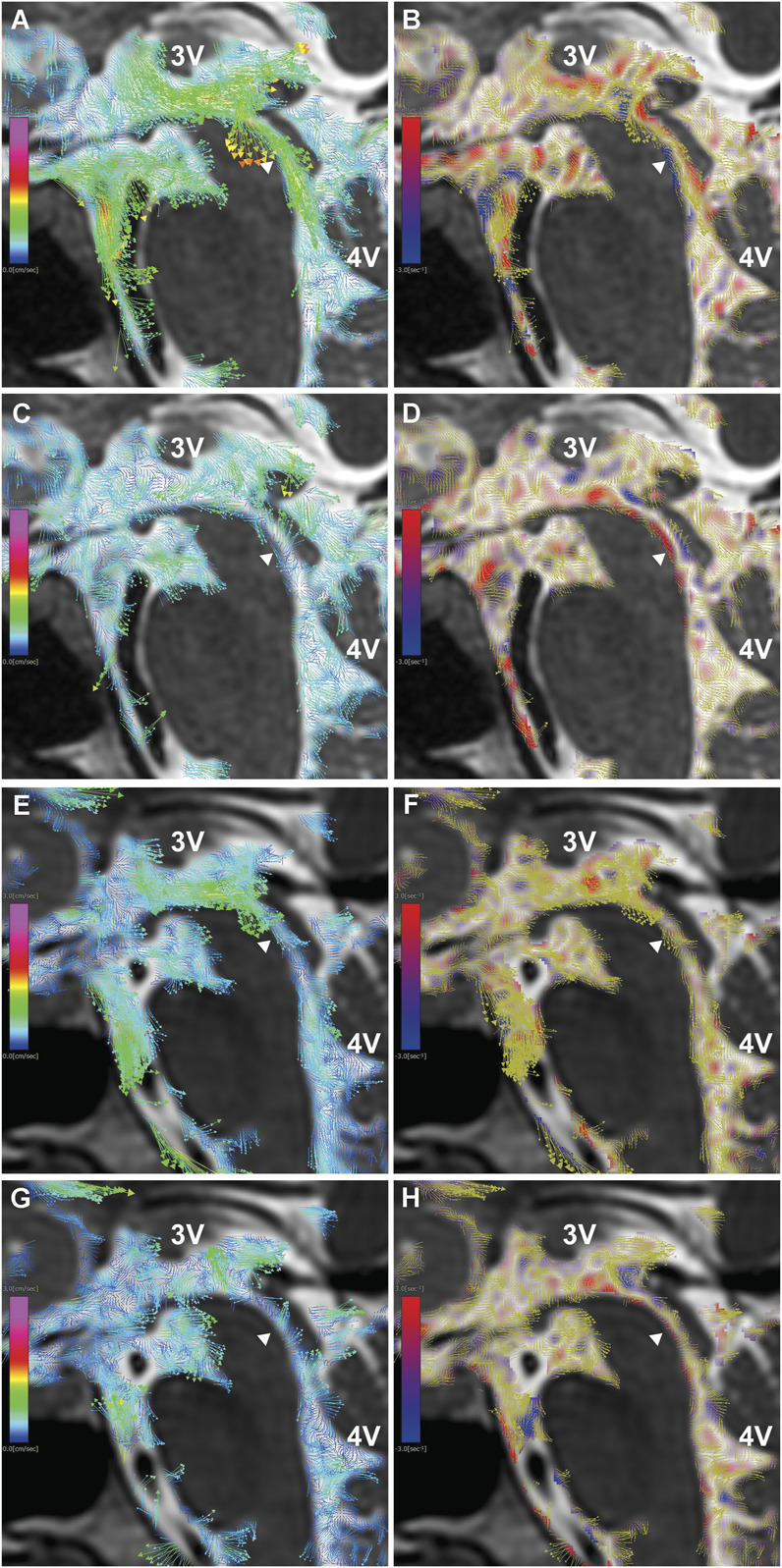
Normal CSF dynamics on 4D flow MRI. 4D flow MRI findings in a healthy 25-year-old man **A**-**D**, and a healthy 48-year-old man **E**-**H**. **A**, **C**, **E**, and **G**, Velocity maps from 2D sagittal views, with colored arrows representing CSF velocity vectors. **B**, **D**, **F**, and **H**, Vorticity maps from 2D sagittal views, showing clockwise vortices in blue, counterclockwise vortices in red, and overlaid velocity vectors in yellow. CSF flow from the 3V into the 4V through the cerebral aqueduct (white arrowhead) was observed. In addition, a short episode of reversed, high-velocity flow from the aqueduct back into the 3V occurred during the same cardiac cycle. During this reversed flow, prominent clockwise (blue) and counterclockwise (red) vortices were observed in the posterior 3V, located behind the interthalamic adhesion (Massa intermedia). 2D, 2-dimensional; 3V, third ventricle; 4D, 4-dimensional; 4V, fourth ventricle; CSF, cerebrospinal fluid.

CSF dynamics around the cerebral aqueduct and the third and 4Vs in the healthy brain, as visualized by 4D flow MRI, exhibited complex patterns with considerable interindividual variability (Figure 1). A large, prolonged downward CSF flow was observed during a single cardiac cycle, with flow vectors extending from the foramina of Monro toward the cerebral aqueduct. In the subsequent phase, short, small upward flow vectors emerged from the aqueduct into the third ventricle (3V). These upward vectors were impeded by the interthalamic adhesion, resulting in turbulence and the formation of superior and inferior vortices. These vortices were synchronized with the next phase of downward flow vectors returning from the 3V into the cerebral aqueduct.

### Case 1: Obstructive Hydrocephalus due to Aqueductal Stenosis Treated With VPS Followed by ETV

**FIGURE 2.**
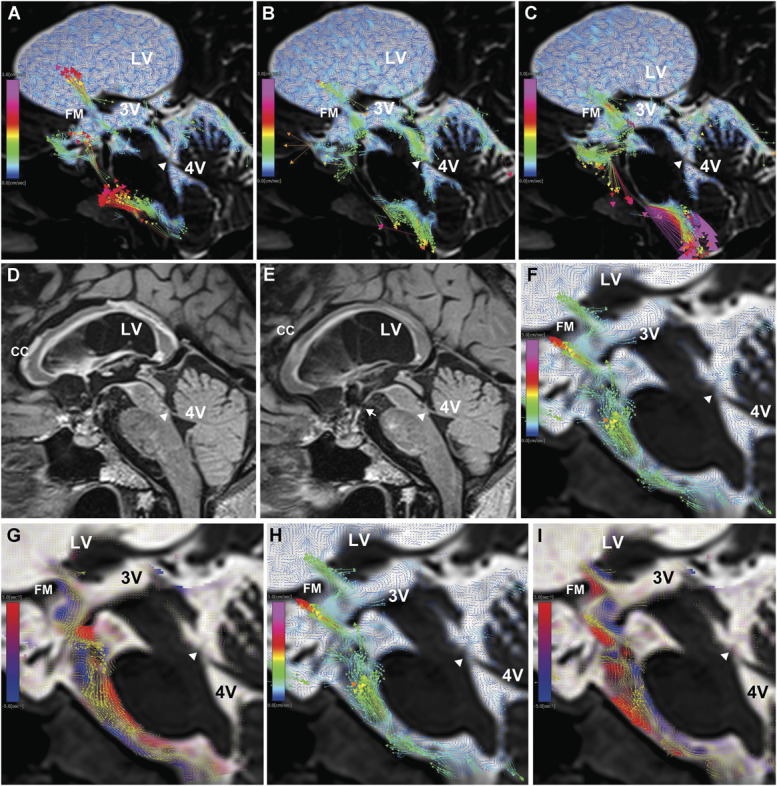
Obstructive hydrocephalus (case 1). **A**-**C**, Sagittal views of 4D flow MRI before treatment. The white arrowhead indicates the obstructed site at the lower end of the cerebral aqueduct, and the colored arrows represent velocity vectors. The FM exhibited the highest bidirectional CSF flow velocity, whereas slow bidirectional flow was also observed within the obstructed cerebral aqueduct. **D**, Sagittal view of 3D FLAIR image taken 4 months after VPS. The CC exhibited widespread high signal intensity across its entire length, as did the upper midbrain and the third ventricular floor. **E** and **F**, Sagittal views of 4D flow MRI after ETV. Rapid CSF flow was observed from the FM into the 3V and through the fenestration in the third ventricular floor (white arrow) into the interpeduncular cistern. During a single cardiac cycle, the flow reversed, showing rapid CSF movement from the interpeduncular cistern into the 3V and from the 3V to the LV through the FM. **G**, Sagittal view of 3D FLAIR image after ETV. Compared with pre-ETV imaging **D**, the widespread high signal intensity in the CC, upper midbrain, and third ventricular floor had improved. **H** and **I**, Sagittal views of 4D flow MRI after ETV. Clockwise vortices are shown in blue, counterclockwise vortices in red, with yellow velocity vectors overlaid. A clockwise vortex was observed in the anterior part of the 3V. 3D, 3-dimensional; 3V, third ventricle; 4D, 4-dimensional; 4V, fourth ventricle; CC, corpus callosum; CSF, cerebrospinal fluid; ETV, endoscopic third ventriculostomy; FLAIR, fluid-attenuated inversion-recovery; FM, foramen of Monro; LV, lateral ventricle; VPS, ventriculoperitoneal shunting.

A 67-year-old woman presented with gait disturbance and mild cognitive impairment. Brain MRI revealed marked enlargement of the lateral ventricles, occlusion of the cerebral aqueduct, and thinning of the corpus callosum. We initially expected a complete absence of CSF circulation in the 3V and cerebral aqueduct. However, 4D flow MRI revealed slow flow around the foramen of Monro and even within the obstructed aqueduct (Figure 2A-2C and Video 1). The patient was diagnosed with late midlife hydrocephalus and underwent VPS, which initially resulted in temporary symptom improvement. However, a few months later, her condition deteriorated rapidly, eventually leading to a profound loss of mobility. The volumes of the lateral and 3Vs were markedly reduced, and 3D fluid-attenuated inversion-recovery imaging revealed high signal intensity surrounding the corpus callosum, third ventricular floor, and midbrain (Figure 2D). She subsequently underwent ETV, which led to a gradual improvement in consciousness and paralysis of the upper and lower limbs, although some neurological deficits persisted. Post-ETV 3D fluid-attenuated inversion-recovery imaging showed an improvement in corpus callosal signal changes (Figure 2E). 4D flow MRI after ETV demonstrated a pulsatile CSF flow continuously extending from the foramen of Monro to the third ventricular floor fenestration (Figure 2F-2I and Video 2).

### Case 2: Obstructive Hydrocephalus due to Aqueductal Stenosis Treated With ETV

**FIGURE 3.**
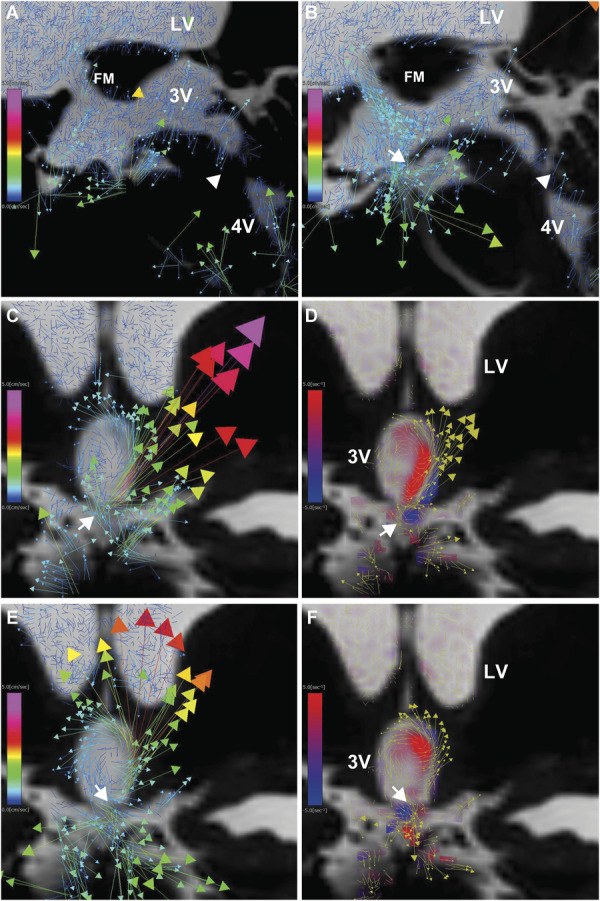
Obstructive hydrocephalus (case 2). **A**, Sagittal view of 4D flow MRI before treatment. The white arrowhead indicates the obstructed site at the lower end of the cerebral aqueduct, and the colored arrows represent velocity vectors. A slight CSF flow velocity was observed around the FM. **B**, Sagittal view of 2D velocity mapping on 4D flow MRI after ETV. Rapid CSF flow was observed from the FM into the 3V and through the fenestration in the 3V floor (white arrow) into the interpeduncular cistern. **C**-**F**, Coronal views of 4D flow MRI after ETV, showing velocity maps with colored arrows indicating velocity vectors **C** and **E**, and vorticity maps **D** and **F**. The vorticity maps show clockwise vortices in blue, counterclockwise vortices in red, and overlaid yellow velocity vectors. CSF entered the 3V through the fenestration in the 3V floor (white arrow), created a strong counterclockwise vortex, and then exited into the interpeduncular cistern. 2D, 2-dimensional; 3V, third ventricle; 4D, 4-dimensional; 4V, fourth ventricle; CSF, cerebrospinal fluid; ETV, endoscopic third ventriculostomy; FM, foramen of Monro.

A 23-year-old man with a history of bacterial meningitis at the age of 2 presented with severe headache and nausea/vomiting, despite a longstanding history of chronic headaches. Brain MRI revealed aqueductal stenosis, significant enlargement of the lateral and 3Vs, and ballooning of the third ventricular floor. The patient underwent ETV, resulting in a marked improvement in symptoms. Preoperative and postoperative 4D flow MRI were performed (Figure 3 and Video 3). Before ETV, 4D flow MRI demonstrated a complete absence of CSF movement in the 3V, indicating CSF stagnation (Figure 3A). After ETV (Figure 3B-3F and Video 4), a large rotational CSF flow emerged in the 3V, as observed on the coronal view of 4D flow MRI.

### Case 3: Juvenile Communicating Hydrocephalus Initially Treated With ETV

**FIGURE 4.**
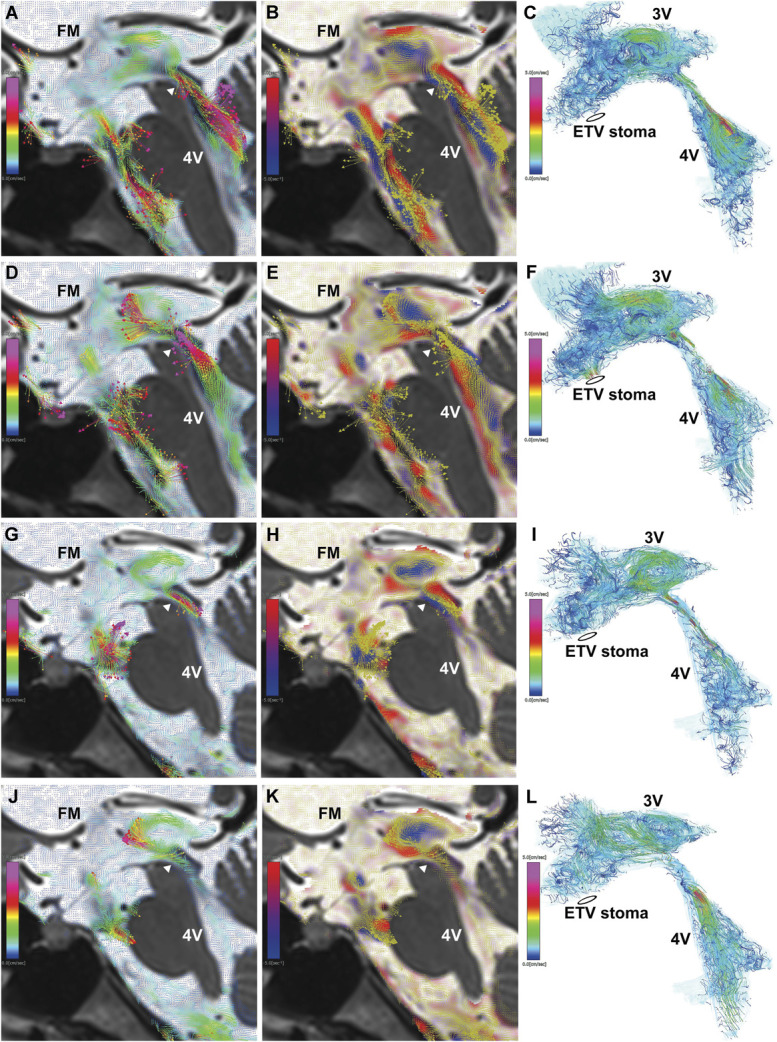
Communicating hydrocephalus (case 3). **A**-**F**, 4D flow MRI after ETV and before VPS. **G**-**L**, 4D flow MRI after VPS. **A**, **D**, and **G**, Velocity maps of 2D sagittal views, with colored arrows indicating velocity vectors. **B**, **E**, and **H**, Vorticity maps of 2D sagittal views, showing clockwise vortices in blue, counterclockwise vortices in red, and overlaid yellow velocity vectors. **C**, **F**, and **I**, Streamlines of CSF motion during a heartbeat in the 3D view; white arrowhead, upper end of the cerebral aqueduct. Rapid CSF flow from the cerebral aqueduct into the 4V and reversed rapid CSF flow from the cerebral aqueduct into the 3V were observed. A clockwise vortex formed in the posterior 3V behind the interthalamic adhesion (Massa intermedia). The fenestration in the 3V floor (ETV stoma) exhibited rapid, repetitive bidirectional flow within a single cardiac cycle. 2D, 2-dimensional; 3D, 3-dimensional; 3V, third ventricle; 4D, 4-dimensional; 4V, fourth ventricle; CSF, cerebrospinal fluid; ETV, endoscopic third ventriculostomy; FM, foramen of Monro; VPS, ventriculoperitoneal shunting.

A 17-year-old adolescent boy developed chronic headaches and central hypersomnia, characterized by a prolonged sleep duration of more than 12 hours per day and frequent daytime napping. He was diagnosed at another hospital with congenital hydrocephalus due to a Blake's pouch cyst and underwent ETV. Although his symptoms temporarily improved postoperatively, they quickly recurred. Follow-up MRI confirmed a patent ETV stoma and improvement in ballooning of the third ventricular floor. However, his neurosurgeons were unable to determine the cause of his persistent symptoms or the next treatment options. Because his symptoms worsened, he developed a mental disorder and was referred to multiple hospitals, ultimately leading to him dropping out of high school. He underwent 4D flow MRI at our institution, which revealed large, violent vortices in the 3V due to intense bidirectional CSF movement in the cerebral aqueduct and ETV stoma (Figure 4A-4F and Video 5). CSF pressure at the lumbar site was elevated (22 cm H_2_O). Although his symptoms did not improve after a CSF tap test, he opted to undergo VPS based on the diagnosis of abnormal CSF dynamics after extensive discussions.

Three months after VPS, his chronic headaches improved first, followed by a gradual improvement in central hypersomnia. Ventricular size slightly decreased, and the violent CSF movements calmed down on 4D flow MRI after VPS (Figure 4G-4L, Videos 6 and 7).

### Case 4: Juvenile Communicating Hydrocephalus Initially Treated With VPS

**FIGURE 5.**
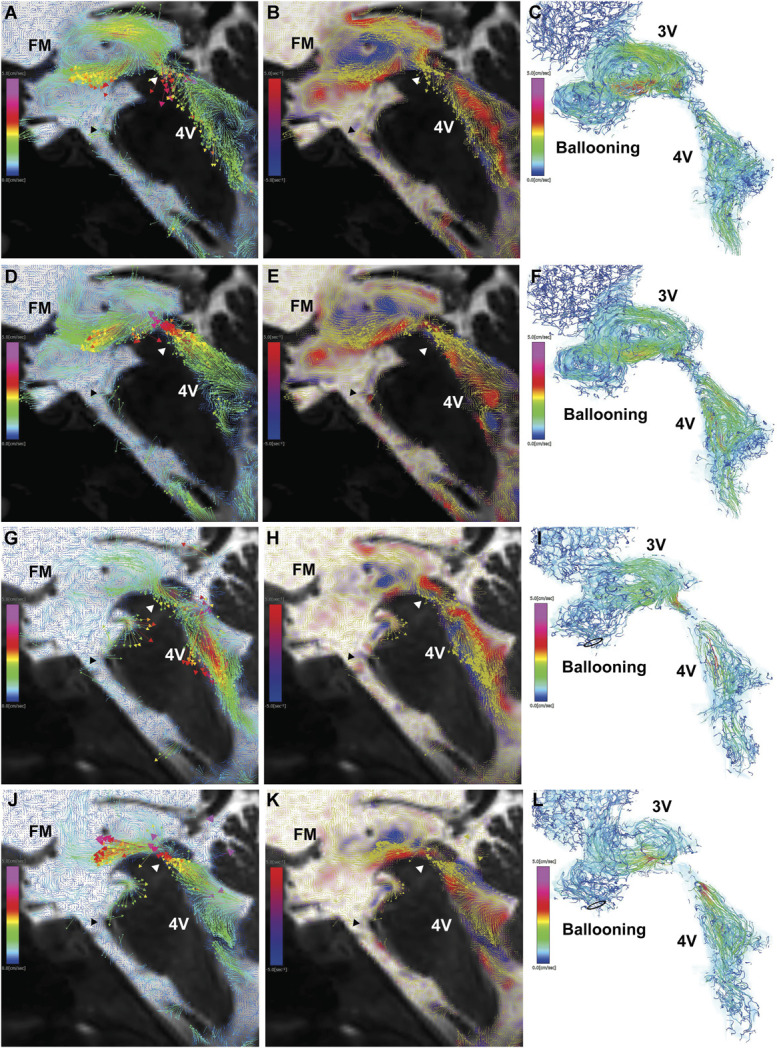
Communicating hydrocephalus (case 4). **A**-**F**, 4D flow MRI before VPS. **G**-**L**, 4D flow MRI after VPS. **A**, **D**, and **G**, Velocity maps of 2D sagittal views, with colored arrows indicating velocity vectors. **B**, **E**, and **H**, Vorticity maps of 2D sagittal views, showing clockwise vortices in blue, counterclockwise vortices in red, and overlaid yellow velocity vectors. **C**, **F**, and **I**, Streamlines of CSF motion during a heartbeat in the 3D view, white arrowhead, upper end of the cerebral aqueduct. Rapid CSF flow from the cerebral aqueduct into the 4V and reversed rapid CSF flow from the cerebral aqueduct into the 3V were observed. A clockwise vortex formed in the posterior 3V, whereas a counterclockwise vortex developed in the anterior 3V with a downward-protruding floor (black arrowhead). After VPS, flow velocity from the cerebral aqueduct into the 4V increased, whereas flow velocity from the cerebral aqueduct into the 3V decreased compared with preshunting imaging. Although ballooning of the 3V floor persisted, the counterclockwise vortex in the anterior 3V had nearly disappeared. 2D, 2-dimensional; 3D, 3-dimensional; 3V, third ventricle; 4D, 4-dimensional; 4V, fourth ventricle; CSF, cerebrospinal fluid; FM, foramen of Monro; VPS, ventriculoperitoneal shunting.

A 21-year-old woman had been experiencing intermittent headaches and vertigo for the past 5 years. Because her headaches worsened, she developed left lower limb motor impairment and balance disturbances, ultimately leading to difficulty walking. She was admitted to the hospital as an emergency, and brain MRI revealed marked enlargement of all ventricles from the lateral to the 4V, along with a flow void sign in the 3V and foramina of Monro, downward ballooning of the third ventricular floor, and abnormal dilation of both the cerebral aqueduct and the foramen of Magendie. After an initial diagnosis of communicating hydrocephalus, she was referred to our institution. 4D flow MRI showed violent oscillating flow and multiple vortices in the third and 4Vs. In addition to a large clockwise vortex in the 3V, another counterclockwise vortex was observed in the downward protrusion of the third ventricular floor (Figure 5A-5F and Video 8). Based on our experience with case 3, we selected VPS as the first-line treatment without performing a CSF tap test. Three months later, her symptoms improved markedly, and she returned to work within a year. Post-VPS 4D flow MRI demonstrated a slight decrease in ventricular size, persistence of third ventricular floor ballooning, and the near-complete disappearance of the counterclockwise vortex in the downward protrusion of the third ventricular floor due to reduced CSF flow velocity from the cerebral aqueduct into the 3V (Figure 5G-5L, Videos 9 and 10).

## DISCUSSION

### Key Result

We presented an overview of the normal CSF flow pattern in the healthy brain and abnormal flow patterns observed in midlife hydrocephalus, a rare form of chronic hydrocephalus in adults,^20^ using 4D flow MRI. In obstructive hydrocephalus with CSF stagnation in the 3V, ETV is generally considered the first-line treatment.^14,15^ The patency of the ETV fenestration can be visually confirmed by the presence of a sustained vortex flow in the 3V on 4D flow MRI. In communicating hydrocephalus, particularly in early midlife hydrocephalus (cases 3 and 4), multiple abnormally intense vortex flows were frequently observed in the huge 3V. Downward bulging of the third ventricular floor may serve as a radiological marker indicating a favorable indication for ETV. However, in cases with highly oscillatory flow and multiple vortices within the 3V, VPS may be preferred, as reducing lateral ventricular volume could help attenuate turbulent CSF motion. These findings highlight the clinical utility of 4D flow MRI in guiding individualized treatment strategies based on comprehensive CSF flow assessment in patients with midlife hydrocephalus.

### Limitations

First, this study was based on visual analysis of CSF flow vectors in a small number of patients with midlife hydrocephalus and healthy controls. Further studies with larger cohorts are necessary to more precisely define abnormal (and normal) CSF dynamics in the 3V. Second, conventional quantitative flow parameters, such as mean flow velocity and stroke volume in the cerebral aqueduct, were not analyzed in this study because considerable interindividual variability in complex CSF motion was observed. In addition, 4D flow MRI captures CSF dynamics during a single cardiac cycle and may not reflect longer-term physiological fluctuations, including those related to respiration.

### Interpretation

In midlife hydrocephalus with marked ventricular enlargement, CSF dynamics often deviate from normal physiological patterns. In obstructive hydrocephalus, CSF stagnation typically results from aqueductal obstruction or stenosis. By contrast, communicating hydrocephalus is characterized by excessive oscillatory flow and turbulence within the third and 4Vs. These abnormal patterns—marked by strong vortices and rapid currents—may disrupt CSF circulation, impair hormonal signaling, and contribute to symptoms such as sleep disturbances and cognitive dysfunction. These observations suggest that failure of CSF dynamics plays a central role in the pathophysiology of both obstructive and communicating forms of midlife hydrocephalus. 4D flow MRI provides valuable insight into these dynamic abnormalities, complementing structural imaging and potentially explaining the variable outcomes of surgical interventions. Incorporating both preoperative and postoperative CSF flow assessments using 4D flow MRI into clinical practice may improve diagnostic precision and support the development of physiology-based, individualized treatment strategies.

### Generalizability

Midlife hydrocephalus is distinguished by lateral ventricular enlargement without accompanying sulcal narrowing or periventricular compression, making diagnosis and management particularly challenging. Recognizing CSF dynamics failure—whether due to aqueductal blockage or excessive intraventricular turbulence—may help explain the condition's clinical heterogeneity. By integrating 4D flow MRI into the diagnostic workflow, clinicians can identify patient-specific flow disturbances and make more informed treatment decisions, such as choosing between ETV and VPS. This approach not only enhances diagnostic accuracy but may also lead to novel therapeutic strategies targeting CSF dynamics, representing a potential paradigm shift in the clinical management of hydrocephalus.

## CONCLUSION

This study demonstrated that 4D flow MRI can visualize abnormal CSF dynamics in midlife hydrocephalus, a rare subtype of chronic hydrocephalus in adults. In obstructive hydrocephalus, third ventricular CSF stagnation due to aqueductal obstruction was clearly identifiable. In communicating hydrocephalus, particularly in younger patients, excessive oscillatory flow and intense vortex formation within the 3V may contribute to symptom development. These findings support the clinical value of 4D flow MRI in identifying pathological CSF dynamics and guiding personalized treatment strategies such as the selection between ETV and VPS. Furthermore, recognizing vortex-related CSF disturbances as a central pathophysiological mechanism offers novel insights into both diagnosis and treatment planning. The results highlight the diagnostic utility of visual flow pattern analysis. Analogous to hemodynamic imaging in cardiology, CSF hydrodynamics assessed by 4D flow MRI may advance clinical decision-making. The proposed concept of CSF dynamics failure offers a novel framework for understanding and managing hydrocephalus, promoting personalized, physiology-based care and improved outcomes.
